# Disentangling direct and indirect effects of experimental grassland management and plant functional-group manipulation on plant and leafhopper diversity

**DOI:** 10.1186/1472-6785-14-1

**Published:** 2014-01-17

**Authors:** Georg Everwand, Verena Rösch, Teja Tscharntke, Christoph Scherber

**Affiliations:** 1 Department of Crop Science, Agroecology, Georg-August-University, Grisebachstrasse 6, 37077 Göttingen, Germany

**Keywords:** Auchenorrhyncha, Management intensity, Mowing, Plant species composition, Forb, Graminoid, Biodiversity experiment, Removal experiment

## Abstract

**Background:**

Plant biodiversity can affect trophic interactions in many ways, including direct bottom-up effects on insects, but is negatively affected by agricultural intensification. Grassland intensification promotes plant productivity, resulting in changes in plant community composition, and impacts on higher trophic levels. Here, we use a novel grassland management experiment combining manipulations of cutting and fertilization with experimental changes in plant functional group composition (independent of management effects) to disentangle the direct and indirect effects of agricultural management on insect herbivore diversity and abundance. We used leafhoppers as model organisms as they are a key insect taxon in grasslands and react rapidly to management changes. Leafhoppers were sampled between May and September 2010 using standardized sweep netting and pan traps.

**Results:**

Plant diversity, functional group composition and management regime in grasslands affected leafhopper species richness and abundance. Higher cutting frequencies directly led to decreasing leafhopper species richness, presumably due to the higher disturbance frequency and the reduction in food-resource heterogeneity. In contrast, fertilizer application had only a small indirect negative effect via enhanced aboveground plant biomass, reduced plant diversity and changes in functional group composition. The manipulated increase in grass cover had contrasting direct and indirect effects on leafhopper species richness: grass cover directly increased leafhopper species richness, but negatively affected plant diversity, which in turn was positively related to leafhopper species richness. In conclusion, insect diversity is driven in complex direct and indirect ways by grassland management, including changes in functional group composition.

**Conclusions:**

The availability of preferred food sources and the frequency of disturbance are important direct and indirect drivers of leafhopper species richness, interacting in complex ways with plant diversity and food resource heterogeneity.

## Background

Grasslands, such as permanent meadows and pastures, cover about 37% of the agricultural area in Europe [1] and harbour much of Europe’s overall biodiversity [2]. Many plant and animal species are restricted to this habitat type [3]. However, agricultural intensification and land-use change have caused major losses in grassland biodiversity [4,5]. Large amounts of fertilizer are applied in grassland to increase yield [6], allowing for earlier and more frequent cuttings in the growing season. Additionally, herbicides may be used to suppress unwanted plant species ( [3]). These management practices greatly affect both plant biomass and species composition. Plant species that are adapted to low nutrient levels and low cutting frequencies are replaced by more competitive, faster-growing species [7,8], thereby altering the invertebrate communities of the grassland as well [9]. Frequent cutting disturbs the vegetation structure, removes food resources and kills many animals [3,10-12].

Here, we present results from a novel grassland management and plant functional group manipulation experiment (GrassMan) [13], combining experimental variations in cutting frequency (two levels, one cut or three cuts) and fertilizer application (two levels, fertilized or unfertilized) with a plant functional group manipulation treatment (three levels), testing the enhancement of grasses or herbs independent of management changes. We focus on insect responses to plant species composition and management intensity. The resulting 12 treatment combinations were replicated six times, resulting in 72 plots laid out experimentally as a Latin rectangle [10].

Other studies on trophic interactions in biodiversity experiments have largely relied on artificially sown gradients in plant diversity [14-16]. To achieve more realistic results, we performed this study in an old grassland and only changed the relative importance of grasses and forbs [17].

We chose leafhoppers, planthoppers and froghoppers (Auchenorrhyncha, hereafter referred to as leafhoppers), as model organisms as they are a highly diverse plant-sucking insect group that has been shown to be strongly influenced by management regime, productivity, vegetation structure and plant species composition [18-20]. Leafhoppers play an important role both as herbivores and as prey for higher trophic levels. Their rapid reaction to changes in management regime makes them highly appropriate for ecological studies such as the one presented here [19]. Nevertheless, they have rarely been studied in this context (but see Hollier *et al.*[21]).

We hypothesize that

(1) Leafhopper abundance and diversity decrease with cutting frequency, as cutting acts as a mechanical disturbance.

(2) Leafhopper abundance and diversity increase with fertilizer application, as this enhances quantity and nutritional quality of available food resources.

(3) Leafhopper abundance and diversity increase under experimental enhancement of graminoid cover, because many species feed preferably on graminoids.

## Results

### Treatment effects on vegetation

**Figure 1:**
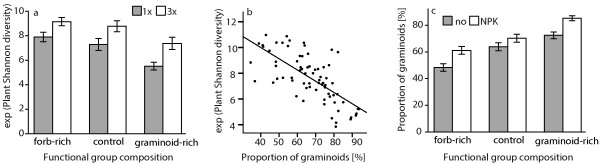
**Changes in plant diversity due to functional group manipulation, fertilization and mowing frequency. (a)** Effects of FG manipulation and mowing frequency on the numbers equivalent (e^H´^) of plant Shannon diversity; only main effects were significant; **(b)** Effects of proportion of graminoids on e^H´^; Spearman’s rho = -0.70; p < 0.001; the regression line represents a linear model (F_1,72_ = 69.43; P < 0.001); **(c)** Effect of FG manipulation and fertilization on the proportion of graminoids. Bars represent the mean; error bars show ±1 SE.

We found 61 plant species, 22 graminoids and 39 forbs (including four legumes). Plant Shannon diversity was positively affected by higher cutting frequency (three times/year) (Figure 1a; Table 1). In addition, FG manipulation significantly affected plant Shannon diversity (Figure 1a; Table 1). Plant Shannon diversity (numbers equivalent, e^H’^) was highest in unfertilized, forb-rich plots with three cuts (9.61 ± 0.35) and lowest in fertilized, graminoid-rich plots with one cut (5.28 ± 0.45; Table 2), but fertilizer effects were generally non-significant (Table 1).

**Table 1:** Sequential F tests of model terms for generalized linear models with plant and leafhopper diversity as response variables

		**Plant Shannon diversity (e**^**H´**^**)**		**Leafhopper Shannon diversity (e**^**H´**^**)**		**% graminoids**	
	Df	F	Pr (>F)	F	Pr (>F)	F	Pr (>F)
Block	5,71	4.70	0.001	4.47	0.002	-	-
FG manipulation	2,71	16.34	<0.001	5.76	0.005	40.87	<0.001
Cutting frequency	1,71	23.06	<0.001	33.03	<0.001	-	-
Fertilizer application	1,71	-	-	-	-	24.53	<0.001
Block:column	6,71	-	-	3.27	0.008	-	-

GLM with Gamma errors (eH´); GLM with logit link (% graminoids).

**Table 2:** Effects of experimental treatments on vegetation parameters (mean ± 1 SE)

**Functional group**	**Cutting frequency**	**Fertilizer application**	**Compr. veg. height [cm]**	**Plant Shannon diversity**	**Proportion grass [%]**	**N**
			**mean (± SE)**	**mean (± SE)**	**mean (± SE)**	
Control	1x	no	10.89 (± 0.29)	6.90 (± 0.68)	68.52 (± 2.75)	6
Forb-rich	1x	no	11.07 (± 0.23)	7.97 (± 0.56)	50.28 (± 2.78)	6
Graminoid-rich	1x	no	10.27 (± 0.11)	5.74 (± 0.48)	75.60 (± 3.82)	6
Control	3x	no	7.87 (± 0.13)	9.44 (± 0.55)	59.32 (± 4.79)	6
Forb-rich	3x	no	7.58 (± 0.12)	9.61 (± 0.35)	46.33 (± 5.07)	6
Graminoid-rich	3x	no	7.79 (± 0.13)	7.57 (± 0.56)	69.27 (± 2.84)	6
Control	1x	NPK	13.36 (± 0.27)	7.65 (± 0.73)	67.95 (± 5.07)	6
Forb-rich	1x	NPK	12.72 (± 0.32)	7.81 (± 0.61)	60.72 (± 5.29)	6
Graminoid-rich	1x	NPK	12.54 (± 0.42)	5.28 (± 0.45)	86.67 (± 2.35)	6
Control	3x	NPK	10.05 (± 0.19)	8.10 (± 0.61)	72.52 (± 3.49)	6
Forb rich	3x	NPK	9.72 (± 0.15)	8.68 (± 0.54)	61.55 (± 3.15)	6
Graminoid-rich	3x	NPK	10.08 (± 0.31)	7.17 (± 0.87)	84.02 (± 3.01)	6

Compr. veg. height = compressed vegetation height.

Plant Shannon diversity was strongly negatively correlated with the proportion of graminoids (Spearman’s rho = -0.70; p < 0.001, Figure 1b, Table 2).

The proportion of graminoids was highest in fertilized, graminoid-rich plots with one cut (86.67 ± 2.35 t ha^-1^) and lowest in unfertilized, forb-rich plots with three cuts (46.33 ± 5.07 t ha^-1^). Generalized linear models with logit-transformed proportion of graminoids (Table 1) showed that the proportion of graminoids increased 0.42-fold with fertilizer application (estimate: 0.42 ± 0.08, t = 4.95, df = 68,P < 0.001). Forb-rich communities had a significantly lower proportion of grasses than control plots (estimate: -0.44 ± 0.1, t = -4.27, df = 68, P < 0.001), and graminoid-rich plots had significantly more grasses than forb-rich plots (estimate: 0.93 ± 0.10, t = 9.04, df = 68, P < 0.001). The positive effect of fertilization on the proportion of graminoids was even higher under FG reduction of forbs (see also Everwand et al. [10]).

### Treatment effects on leafhoppers

In total, we caught 6497 adult leafhopper specimens from 36 species (Additional file 1: Table S4). Twenty-eight species (86.5% of the total abundance) were graminoid-feeders, and eight species were forb-feeders [22]. The four most common species were *Arthaldeus pascuellus* (Fall.) with 67.9% of total abundance, *Philaenus spumarius* (L.) (10.7%), *Streptanus sordidus* (ZETT.) (7.6%) and *Macrosteles viridigriseus* (EDWARDS) (2.7%).

**Figure 2:**
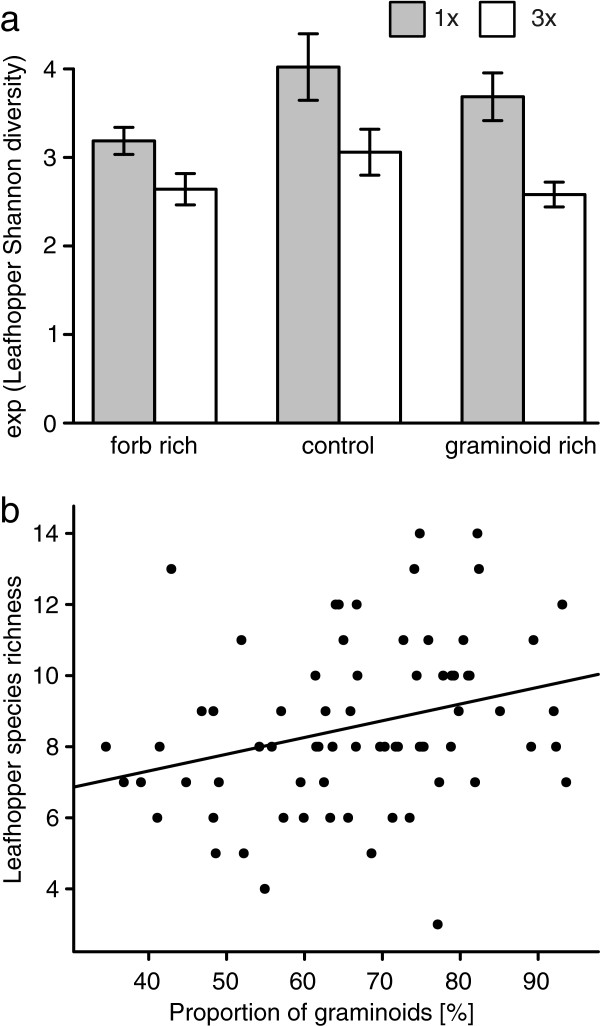
**Changes in leafhopper diversity due to functional group manipulation and mowing frequency. (a)** Effects of FG manipulation and cutting frequency on the numbers equivalent of Shannon diversity of leafhoppers (e^H´^); **(b)** Positive relationship between leafhopper diversity [species per plot] and proportion of graminoids [%] (Spearman’s rho = 0.35; p = 0.003); the regression line represents a linear model (F_1,72_ = 6.56; P = 0.013). Bars represent the mean; error bars show ±1 SE.

Leafhopper Shannon diversity (e^H´^) was highest in the most natural and least disturbed plots (one cut, no herbicide) with an average of 4.02 ± 0.38 species per plot (Figure 2a, Additional file 2: Table S2). A consistently negative effect of three cuts per year (Figure 2a, Table 1, Additional file 2: Table S2) and of FG manipulation (Figure 2a, Table 1, Additional file 2: Table S2) was observed. Fertilizer application had no direct effect on leafhopper Shannon diversity. Untransformed leafhopper species richness was positively affected by the proportion of graminoids (F_1,72_ = 6.56; P = 0.013; Figure 2b). For a numerical summary of effects on leafhoppers, see Additional file 3: Table S5.

### Direct and indirect treatment effects on vegetation

**Figure 3:**
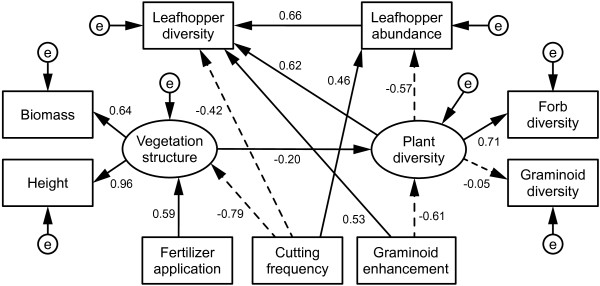
**Structural equation model showing indirect and direct treatment effects on leafhoppers.** The graph shows the minimal adequate structural equation model with N = 72, X^2^ = 18.7, P = 0.664, 22 degrees of freedom and a root mean squared error of approximation of 0.00 (90% confidence interval = [0, 0.081]). Rectangles represent observed variables (organism abundance and diversity = species richness), ellipses represent latent variables. Solid (dashed) arrows indicate positive (negative) relationships among variables. Numbers next to arrows and boxes are standardized path coefficients. Design variables were specified as numeric variables. Fertilizer application: no fertilizer = 0; NPK-fertilizer = 1; cutting frequency; one cut per year = 0; three cuts per year = 1; and Graminoid enhancement: graminoid-reduced = -1; control = 0; forb-reduced = 1.

Structural equation modeling (Figure 3) showed that an increasing proportion of graminoids, due to FG manipulation, reduced plant diversity (standardized path coefficient ß = -0.61). Plant diversity (latent variable) was mainly driven by forb diversity (ß = 0.71). Fertilizer application and cutting frequency jointly influenced the latent variable “plant productivity”: fertilization increased plant biomass and height, while cutting reduced both. Finally, higher plant productivity negatively influenced plant diversity (ß = -0.2). Notably, alternative pathways, e.g. arrows from plant diversity to plant productivity, were not supported by our data.

### Direct and indirect effects of treatments and vegetation on leafhoppers

Our structural equation model (Figure 3) indicated that there were both direct and indirect effects of treatments on leafhopper abundance and species richness.Plant functional group manipulation was the most important direct driver of leafhopper species richness (ß = 0.53): In forb-rich communities, leafhopper species richness was lowest, while it was highest in grass-rich communities. In contrast, a higher percentage of grasses affected plant diversity negatively, while the number of plant species (latent variable “plant diversity”) had a positive effect on leafhopper species richness (ß = 0.62, see Figure 3). Cutting frequency had a negative effect on leafhopper species richness (ß = -0.42), whereas fertilizer application exhibited no effect on leafhopper species richness. Finally, leafhopper species richness was strongly related to leafhopper abundance (ß = 0.66).

In addition to these direct effects, the treatments had indirect effects on leafhopper abundance and species richness. This was mediated by changes in plant productivity and plant diversity. An inspection of the standardized total effects (Table 3) shows that FG manipulation was the most important driver of leafhopper species richness (total effect ϵ = 0.379). In addition, the latent variable “plant diversity” positively affected leafhopper species richness (ϵ = 0.249).

**Table 3:** Standardized total effects from structural equation model (ϵ), combining indirect and direct effects

	**Fertilizer application**	**Cutting frequency**	**FG manipulation**	**Plant productivity**	**Plant species richness**	**Leafhopper abundance**
**Plant productivity**	0.587***	-0.793***				
**Plant species richness**	-0.117	0.158	-0.613***	-0.200		
**Leafhopper abundance**	0.067	0.368***	0.348***	0.113	-0.568**	
**Leafhopper diversity**	-0.029	-0.080*	0.379*	-0.050	0.249	0.659**

* = P< 0.05;** = P< 0.01;*** = P< 0.001.

**Table 4:** Effects of experimental treatments on leafhopper Shannon diversity, abundance and species richness (mean ±1SE)

**Herbicide**	**Cutting frequency**	**Leafhopper Shannon diversity**	**Leafhopper abundance**	**Leafhopper species richness**	**N**
Control	1x	4.02 (± 0.38)	72.58 (± 7.33)	9.58 (± 0.83)	12
Forb-rich	1x	3.19 (± 0.15)	60.75 (± 8.69)	7.08 (± 0.34)	12
Graminoid-rich	1x	3.69 (± 0.27)	87.00 (± 10.67)	9.67 (± 0.38)	12
Control	3x	3.06 (± 0.26)	110.58 (± 9.03)	9.42 (± 0.71)	12
Forb-rich	3x	2.64 (± 0.18)	80.00 (± 9.81)	7.00 (± 0.41)	12
Graminoid-rich	3x	2.58 (± 0.14)	130.50 (± 19.31)	8.75 (± 0.80)	12

## Discussion

The combination of management measures such as cutting and fertilizer application with the manipulation of functional group composition and their interactions led to contrasting sward types, ranging from nutrient poor, forb-dominated plots harbouring a greater diversity of plants to highly productive, graminoid-dominated plots with lower plant diversity. This had both direct and indirect effects on leafhopper species richness and abundance. Leafhopper species richness profited directly from both a higher cover of graminoids (due to herbicide-induced reduction of forbs) and from a lower cutting frequency. However, an indirect (negative) effect on leafhopper species richness was caused by the higher proportion of graminoids due to forb reduction and management intensification, which both had a negative impact on plant diversity.

Plant diversity had a negative effect on leafhopper abundance in our study, likely because the most abundant leafhopper species such as *Arthaldeus pascuellus* were grass feeders [22]. Plant diversity was driven by forbs, whereas productivity was driven by graminoids, which benefit from fertilizer application. Therefore, fertilizer application led to lower plant diversity but higher amounts of harvestable aboveground biomass.

The grassland in the experimental field site has been used for cattle grazing and hay making for at least a century. We therefore also expect the leafhopper community to be adapted to the long-term managed, grass-dominated vegetation, which may have led to the selection of the dominant species in the pool associated with grasses [22].

The increase in plant Shannon diversity (e^H´^) with graminoid reduction, the decrease in plant Shannon diversity with forb reduction, as well as the strong decrease in plant Shannon diversity with increasing proportion of graminoids highlights the impact of the functional group manipulation on plant diversity and composition and the strong contribution of forbs to plant species richness. Some graminoid species, such as *Agrostis capillaris, Festuca rubra* and *Dactylis glomerata*, play a dominant role in productivity in our study, resulting in a negative species richness–biomass relationship [23].

Plant diversity was particularly high under a higher frequency of three cuts per year, confirming that cutting can increase plant species diversity due to removal of nutrients from the soil [24]. However, a moderate frequency of two cuts per year may improve plant species richness in our experimental field site even more, because the disturbance rate is lower but removal of nutrients is still high [25]. This nutrient limitation only occurs when no fertilizer is applied and the harvested AGB is removed. Recent studies have shown that such grasslands may harbour a higher species number and proportion of forbs [26]. This is in line with our finding of a higher number of plant species (mainly driven by forbs) under the regime of three cuts per year.

When fertilizer is applied, many herbs cannot efficiently use higher nitrogen inputs [25] and are out-competed by more competitive species [27], which are mainly graminoids [28]. However, disturbance events such as mowing create niches for weaker competitors [29].

Similar to Morris [30], we observed a negative direct effect of higher cutting frequency on leafhopper species richness, but a positive effect on leafhopper abundance. However, we did not observe any significant direct responses of leafhopper species richness, abundance or Shannon diversity (e^H´^) to fertilizer application, which are often described in the literature (e.g. [31]).

As indicated by our structural equation model, fertilizer application had a weak indirect negative effect on leafhoppers, mediated by the strong increase in aboveground primary production and its negative association with plant species diversity.

NPK fertilizer application has been shown to result in a strong increase in aboveground biomass production [32] along with an increase in the proportion of graminoids [29] and a decrease in plant species diversity [33]. In our study, this may have been due to the higher tolerance of the dominant graminoid species (*Dactylis glomerata*, *Festuca rubra*) to high cutting frequencies and their faster re-growth capacity after cutting, especially under fertilizer application [34].

Many generalist leafhoppers can benefit from the improved performance of some graminoids under more frequent cutting, as the opposing effect of cutting frequency on leafhopper abundance (positive) and leafhopper species richness (negative) in the path diagram shows. Some very abundant species (e.g. *Arthaldeus pascuellus*, *Streptanus sordidus*, *Deltocephalus pulicaris*), which are generalists on graminoids [22], show a clear preference for plots cut three times per year.

The negative direct effect of higher cutting frequency on Shannon diversity (e^H´^) and species richness of leafhoppers indicates that the majority of leafhopper species found within our study site showed a clear preference for plots cut only once a year, which is in line with findings of other studies [31,35]. This preference of leafhoppers for plots cut only once a year [31,35] is also supported by our finding of a negative impact of higher cutting frequency on vegetation height and biomass. This shows that reduced disturbance rate results in larger amounts of food resources and shelter due to higher vegetation and aboveground biomass. Higher vegetation and aboveground biomass were shown by Kőrösi et al. [36] to have a positive effect on leafhopper abundance and species richness. We expect the same to apply here, since lower vegetation height and biomass on plots cut three times per year also implies a lower amount of available food resources and therefore directly results in lower leafhopper species richness.

The strong positive direct effect of the herbicide-induced increase of graminoids on leafhopper species richness can be explained by the preference for graminoids displayed by most leafhopper species found in our study (see Additional file 4: Figure S3 and Additional file 1: Table S4) which is in accordance with other studies [37].

Higher plant diversity has also been shown to lead to more diverse leafhopper communities [19], which, in our study, is owed to less common but more specialized species (e.g. *Acanthodelphax spinosa*, *Cicadula persimilis*, *Conomelus anceps*).

The herbicide treatment was applied only once in 2008. Nevertheless, this manipulation of plant functional group composition was efficient, since plant diversity and functional group composition were clearly affected even three years after the treatment.

Although most leafhopper species are mobile [18,38] and our plots were small, the differences in management and plant functional group composition caused species sorting even on a small scale, which has also been found to be the case for highly mobile bees and wasps [39].

Our experimental manipulation of established grassland shows very strong design effects on vegetation, therefore we expect the direct and indirect effects of management regime and vegetation parameters on leafhoppers to be even stronger in larger and unconnected areas [40]. Our findings also indicate that leafhoppers prefer certain microhabitats within a defined community.

To gain a deeper understanding of the interactions behind the design effects (cutting frequency, fertilizer application and FG manipulation), we included vegetation parameters, such as vegetation height, harvested peak biomass and plant species richness (for forbs and graminoids separately) in our structural equation model.

The strong direct negative effect of higher cutting frequency and the positive direct effect of higher proportion of graminoids (due to FG manipulation) on leafhopper species richness (and also Shannon diversity, e^H´^) may be explained by several causes: (i) Plant diversity was greater with herbicide-induced reduction of graminoids, higher cutting frequency and without application of NPK-fertilizer, since it was mainly driven by forbs. But species richness of graminoids, which are the preferred food source for the majority of the leafhopper species caught [22] did not increase. Therefore, a higher proportion of graminoids indirectly reduced leafhopper species richness via its negative effect on plant diversity, but also had a direct positive effect due to greater availability of the preferred food source.

(ii) Cutting is a disturbance event and reduces food resource heterogeneity. The higher its frequency, the higher the direct impact on leafhoppers, since many leafhoppers are killed and removed during the process of harvesting [11]. This is comparable to the negative effects of cutting on slug abundance within the same experiment [10].

(iii) The lower vegetation height and biomass on plots cut three times per year also implies a lower amount of available food resources and therefore results in lower leafhopper species richness.

The proportion of graminoids increased with fertilizer application and also with application of forb-specific herbicides. This increased proportion of graminoids may explain the negative effect of vegetation biomass and height on plant diversity, as indicated by our structural equation model, demonstrating how a continuously managed agricultural system results in a high-yield but low-diversity system. This can be associated with less resilience towards sudden perturbation, microclimatic change and invasions [29].

## Conclusions

We have shown that contrasting sward types may result from direct and indirect interactions among management regimes (cutting frequency and fertilizer application) and the manipulation of functional group composition.

Leafhopper species richness increased indirectly in nutrient-poor, forb-dominated plots, since these harboured a greater diversity of plants and therefore greater food-resource heterogeneity. On the other hand, leafhopper species richness increased directly with higher graminoid cover in highly productive plots with lower plant diversity, likely due to a greater availability of graminoids as a preferred food source for many leafhopper species.

With our novel approach of combining variation in cutting and fertilization with a manipulation of plant functional group composition (independent of management effects), we were able to disentangle the complexity of direct and indirect effects within a 100-year old, moderately species-rich and continuously managed grassland, based on the example of the easily accessible group of leafhoppers.

This study allows insights into the effects of grassland management on plant diversity, productivity and functional group composition and the trophic links and feedbacks on leafhopper species richness and abundance. Furthermore, we show that - apart from food resource heterogeneity represented by plant diversity - the availability of preferred food sources and the frequency of disturbance are important drivers of leafhopper species richness, but interactions between biodiversity and management are highly complex.

## Methods

### Description of study site

This study was performed in 2010 as part of the “GrassMan”-Experiment [13] near Neuhaus (Solling) in the Solling Mountains in Northern Germany (51°440 N, 9°320 E, 490 m a.s.l.).

Prior to the start of the experiment the study site was a nutrient poor, moderately wet Lolio-Cynosuretum grassland with high abundances of *Agrostis capillaris* (L.), *Festuca rubra* (L.), *Rumex acetosa* (L.), *Veronica chamaedrys* (L.) and *Ranunculus repens* (L.) [13].

Mean annual precipitation is 1028 mm and mean annual temperature is 6.9°C (Deutscher Wetterdienst, 1961–1990, station Holzminden-Silberborn, 440 m a.s.l.). In 2010, the year of the study, mean annual temperature was 8.0°C and annual precipitation was 1110 mm. The dominant soil type in the experimental area is a shallow (40–60 cm), stony Haplic Cambisol, developed on sediments of loess on the Middle Bunter (Triassic sandstone) formation with a loamy silt texture [41].

### Study design

The experiment was established in 2008 in a permanent, formerly extensively used, cattle-grazed grassland. It was laid out as a three-factorial Latin rectangle [42] with the following factors (Additional file 5: Figure S1): (i) plant functional group manipulation (three levels) using herbicides, (ii) fertilizer application (two levels) and (iii) cutting frequency (two levels), resulting in twelve treatment combinations.

To manipulate plant functional group presence, we applied (i) a combination of the forb-specific herbicides Fluroxypyr (Starane; Dow AgroSciences, Munich, Germany; 3 L ha^-1^) and Mecoprop-P (Duplosan; KV, Du Pont de Nemours, Neu-Isenburg, Germany; 3 L ha^-1^) or (ii) the graminoid-specific herbicide Select 240 EC (Stähler Int., Stade, Germany; 0.5 L ha^-1^), resulting in three levels of plant diversity: (i) forb-reduced (=graminoid-rich), (ii) graminoid-reduced (=forb-rich) and (iii) control. Herbicides were applied once in June 2008 (a “pulse” experiment sensu Bender et al. [43]).

In 2009 and 2010, plots were fertilized with N (Calcium ammonium nitrate N27: 13.5% NH_4_-N, 13.5% NO_3_-N, 4% MgO, 6% Ca) at two equal doses (2 × 90 kg ha^-1^) in April and May/June; in addition, fertilized plots received 30 kg P ha^-1^ and 100 kg K ha^-1^ in early June (Thomaskali®, 8% P_2_O_5_, 15% K_2_O, 20% CaO).

**Figure 4:**
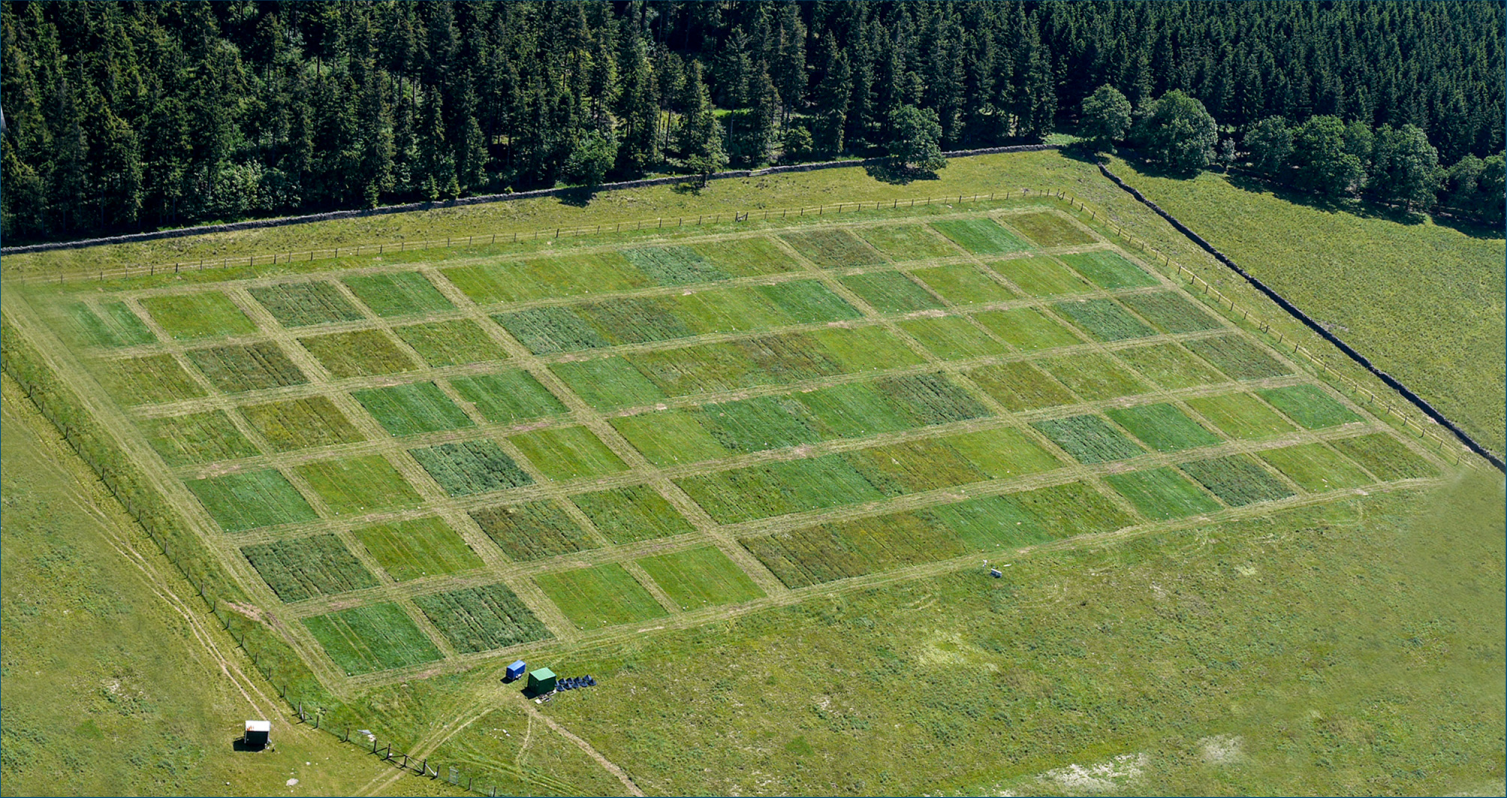
**Aerial photograph of the GrassMan experiment.** Photograph taken on 13th June 2009 by Laura Rose. The horizon was levelled using the ruler tool in Adobe Photoshop CS6.

Control plots were not fertilized. Plots were cut either once (in July) or three times a year (May, July, September) using a Haldrup® forage combine harvester (INOTEC Engineering GmbH, Ilshofen, Germany) at a cutting height of 7 cm. The resulting twelve treatment combinations (equalling one block of the Latin rectangle; see Everwand et al. [10], Figure 4 and Additional file 5: Figure S1) were arranged randomly and replicated six times, resulting in 72 plots. Each plot was a 15 × 15 m square surrounded by at least 3 m of frequently cut grass between plots, and 5 m between blocks.

Plant functional groups were not entirely removed, but target plant species were strongly reduced in abundance. Plant functional groups slowly recovered following herbicide application, but all FG manipulation treatments significantly affected vegetation parameters, such as compressed vegetation height, harvested biomass, functional group composition and plant species richness. More details on the experimental design, setup and treatment effects on vegetation can be found in Petersen et al. [13,44] and Rose et al. [23,45,46].

### Leafhopper sampling

Leafhoppers were sampled using two methods: i) by sweep netting (Heavy Duty Sweep Net, 7215HS, BioQuip, diameter: 38 cm), while walking a circular transect with a diameter of 8 m around the centre of each plot (30 sweeps each), in dry weather on two occasions (at the beginning of July and at the end of August 2010). Transects length was approximately 20 m, and there was a distance of at least 4 m to the edge of each plot. In addition, ii) we sampled leafhoppers by placing two transparent pan traps, containing an ethylene glycol/water mixture (1:3), 1 m apart, near to the centre of each plot. Pan traps were about 5 cm above vegetation height and were active for one week in five time intervals in 2010 (end of June, mid-July, early August, mid-August, end of August).

The specimens caught with both methods were transferred into ethanol (70% vol.) separately and identified to species level in the laboratory using Biedermann & Niedringhaus [47] and Kunz et al. [48]. One species with woody host plants was excluded, as we assumed that it had been swept off its host tree by wind and was not a true member of the grassland fauna. Species whose larvae used herbs or grasses as host plants and whose imagines fed on trees were also included in the analysis.

For female specimens of several genera, identification to species level is not possible (e.g. *Psammotettix*) [47,48]. Thus, if male specimens were present, female specimens were assumed to belong to the same species. If not, they were only identified to genus level. If males of more than one species of a genus were present, the proportion of females was assumed to mirror that of males.

We found no interaction effects of the two sampling methods with the management variables (cutting frequency, fertilizer application) on leafhopper species richness (see e.g. Figure 4, Additional file 6: Table S1 and Additional file 7: Figure S2). In addition, vegetation height (a proxy for vegetation density) did not affect the number of leafhoppers caught by sweep netting.

We therefore pooled the data of both methods, which allowed us to cover the growing season of 2010 from early May until late September. For all diversity assessments, we used species richness, Shannon’s diversity index (H’) or its numbers equivalent exp(H’) [49].

### Assessment of vegetation parameters

Because our treatments are likely to have affected plant productivity and vegetation structure, possibly indirectly affecting leafhopper species richness, we additionally measured a series of vegetation parameters:

(i) We conducted vegetation surveys on two quadrates, each of 1 m^2^ size, twice (in May before the first harvest and also again in August) on each plot. We recorded the percentage of cover, proportional yield of each species [50], plant species richness, functional group composition and presence-absence data of the functional groups (graminoids and forbs).

(ii) Plant aboveground biomass (AGB) was estimated as follows: First, during harvest, fresh weight of two 1.50 × 15 m strips per plot was measured using the harvester’s built-in scale.

To determine the water content of this sample, we took four subsamples that were homogenized, weighed, dried for 48 h at 65°C and subsequently weighed again. We then multiplied fresh weight by water content to obtain the total aboveground dry biomass (t ha^-1^) for every plot.

(iii) Proportions of graminoids and forbs (%) were determined from the vegetation surveys (derived as described above). Harvest was performed on all plots once a year in the end of June and additionally in mid-May and mid-September for the 3-cut treatment [13].

(iv) Compressed sward height (cm) was measured using a rising plate meter according to Castle [51] and the average value of 25 measures per plot was calculated. This was performed every 2-3 weeks, resulting in eleven time points throughout the growing season of 2010.

### Statistical analysis

Data were analysed using the statistical software package R (version 2.15.2) [52]. In addition, we performed structural equation modelling using AMOS 20.0 (SPSS, Inc.). Treatment effects on vegetation and leafhoppers were assessed using generalized linear models (GLMs; [53]).

Models contained row- and column effects (fitted as factors, column was nested within block), sward composition (factor with three levels), cutting frequency and nutrient input (two levels each) with up to two-way-interactions. For abundance data (e.g. Table 4) we used quasipoisson GLMs, for proportion data LMs with a logit link [54,55] and for exp(H’) we used GLMs with Gamma errors and an inverse link. Corresponding alternative models (e.g. quasipoisson or Gamma with log link) had higher residual deviance and were therefore not considered.

Continuous response variables (e.g. biomass or vegetation height) were log-transformed and analyzed using GLMs with an identity link. For each response variable in turn, maximal models containing all possible terms were manually simplified into models containing fewer explanatory variables. We compared the resulting nested models using F-tests (and Chi^2^ for quasipoisson models), until a minimal adequate model that only contained significant effects was obtained.

Significance of terms was assessed in two ways: (i) each parameter estimate from linear models was compared to zero using marginal t-tests; and (ii) terms in the models were additionally tested by sequential addition to a null model (sequential analysis of deviance tables; Additional file 8: Table S3).

In addition to traditional GLM-based analyses, we employed structural equation models (SEMs), allowing us to test more complex hypotheses on indirect effects of treatments, plant productivity and plant diversity on leafhoppers [56-58]. SEMs are particularly well suited for experimental contexts, i.e. where some variables are deliberately manipulated experimentally [59]. Furthermore, SEMs “can be used to develop accurate and meaningful final multiple regression models when collinearities among explanatory variables are thought to be present” [60], which was clearly the case for the vegetation properties measured here.

SEMs contained all three treatment variables, as well as latent variables [56] for plant productivity and plant diversity. For the SEMs we specified our design variables as numeric variables as follows:

Fertilizer treatment: no fertilizer = 0; NPK-fertilizer application = 1

Cutting frequency: one cut/year = 0; three cuts/year = 1

FG manipulation: FG graminoid-reduced = -1; FG control = 0; FG forb-reduced = 1

The sorting of FG manipulation was according to its effect on plant diversity and proportion of graminoids (see Figure 1). Plant productivity had two indicator variables: harvested aboveground biomass in July (AGB, t ha^-1^), and average compressed sward height. Plant diversity had the indicator variables “forbs” and “graminoids”; since only four legumes species (*Lotus corniculatus*, numeric variables as follows*L. pedunculatus*,*Trifolium repens*, *Lathyrus pratensis*) were present in a very low cover on 61 plots only, and none of the leafhopper species found had been categorized as preferentially feeding on legumes, we did not take legumes into account separately for the SEMs. Leafhopper abundance and species richness were taken separately (instead of (e^H`^) Shannon diversity) for the SEM to identify effects of design variables and vegetation parameters on leafhoppers.

## Competing interests

The authors declare that they have no competing interests.

## Authors’ contributions

GE, TT and CS designed the study. GE sampled leafhoppers in the field. GE and VR determined leafhopper species. GE and CS did all statistical analyses. All authors contributed to manuscript drafting, writing and revisions. All authors read and approved the final manuscript.

## Supplementary Material

Additional file 1: Table S4 Species list of the Auchenorrhyncha within the GrassMan experiment with food preferences according to Nickel & Remane (2002) and their abundance. Click here for file

Additional file 2: Table S2 ANOVA-table: Leafhopper species richness vs. Design. Click here for file

Additional file 3: Table S5 Mean and standard error of leafhopper responses to experimental design (treatments). Click here for file

Additional file 4: Figure S3 Box plot showing the effects of herbicide application and cutting frequency on Shannon diversity of grass specialist leafhopper. Click here for file

Additional file 5: Figure S1 Experimental design of the Grassman Experiment, showing the Latin rectangle of 12 treatments in 6 replications. Gra- = graminoid reduced plots (=forb enhanced); Forb- =forb reduced (=graminoid enhanced); Con = Control (no herbicide application). The grey area around and between the plots is mown monthly. Plot size 15 m x 15 m, space between plots 3 m, between blocks 5 m Click here for file

Additional file 6: Table S1 F and p-values of glm’s testing for significant effects of sampling method in combination with design treatments. Click here for file

Additional file 7: Figure S2 Comparison of the two different sampling methods in combination with functional group manipulation and cutting frequency. (a) leafhopper species richness; (b) leafhopper Shannon diversity (eH´); (c) leafhopper abundance. Click here for file

Additional file 8: Table S3 ANOVA-table: Leafhopper abundance vs. Design. Click here for file
