# Supplementary material for: Disentangling direct and indirect effects of experimental grassland management and plant functional-group manipulation on plant and leafhopper diversity
Source: BMC Ecol. 2014 Jan 17;14:1. doi: 10.1186/1472-6785-14-1 (PMC3945068; doi:10.1186/1472-6785-14-1)
Supplement: Additional file 1: Table S4 — Species list of the Auchenorrhyncha within the GrassMan experiment with food preferences according to Nickel & Remane (2002) and their abundance. [file 1472-6785-14-1-S1.pdf]

Table S4: Species list of the Auchenorrhyncha within the GrassMan experiment with food preferences according to Nickel & Remane (2002) and their abundance.

Species	feeding preferences	Abundance
Acanthodelphax spinosa	graminoids	14
Anoscopus flavostriata	graminoids	11
Anoscopus serratulae	graminoids	5
Anoscopus spec.	graminoids	10
Aphrodes makarovi	forbs	8
Aphrophora alni	forbs	4
Arthaldeus pascuellus	graminoids	4414
Balclutha punctata	graminoids	54
Cicadella viridis	graminoids	8
Cicadula persimilis	graminoids	128
Colobotettix morbillosus	forbs	1
Conomelus anceps	graminoids	83
Cosmotetix costalis	graminoids	1
Criomorphus albomarginatus	graminoids	1
Deltocephalus pulicaris	graminoids	162
Elymana sulphurella	graminoids	6
Errastunus ocellaris	graminoids	5
Eupteryx aurata	forbs	2
Eupteryx vittata	forbs	1
Evacanthus interruptus	forbs	1
Javesella dubia	graminoids	58
Javesella pellucida	graminoids	29
Kosswigianella exigua	graminoids	1
Macrosteles viridigriseus	graminoids	174
Macustus grisescens	graminoids	1
Megadelphax sordidula	graminoids	1
Megophtalamus scanicus	forbs	34
Mirabella albifrons	graminoids	1
Neophilaenus lineatus	graminoids	24
Philaenus spumarius	forbs	698
Psammotettix confinis	graminoids	49
Stenocranus minutus	graminoids	4
Stiroma bicarinata	graminoids	1
Streptanus sordidus	graminoids	492
Xanthodelphax stramineus	graminoids	5
Zyginidia scutellaris	graminoids	6
