# Supplementary material for: Disentangling direct and indirect effects of experimental grassland management and plant functional-group manipulation on plant and leafhopper diversity
Source: BMC Ecol. 2014 Jan 17;14:1. doi: 10.1186/1472-6785-14-1 (PMC3945068; doi:10.1186/1472-6785-14-1)
Supplement: Additional file 2: Table S2 — ANOVA-table: Leafhopper species richness vs. Design. [file 1472-6785-14-1-S2.pdf]

Table S2: Sequential analysis of variance table of generalized linear model on leafhopper species richness vs. design variables

	Df	Deviance	Resid.Df	Resid.Dev	F	Pr(>F)
NULL	71.000	19.347				
as.factor(block)	5.000	2.722	66.000	16.625	3.788	0.005
FG manipulation	2.000	1.505	64.000	15.120	5.237	0.008
cutting frequency	1.000	4.284	63.000	10.836	29.813	<0.001
as.factor(block):col	6.000	2.571	57.000	8.265	2.982	0.013
