# Supplementary material for: Disentangling direct and indirect effects of experimental grassland management and plant functional-group manipulation on plant and leafhopper diversity
Source: BMC Ecol. 2014 Jan 17;14:1. doi: 10.1186/1472-6785-14-1 (PMC3945068; doi:10.1186/1472-6785-14-1)
Supplement: Additional file 3: Table S5 — Mean and standard error of leafhopper responses to experimental design (treatments). [file 1472-6785-14-1-S3.pdf]

Table S5: Mean and standard error of leafhopper responses to experimental design variables (treatments)

**Sward**	**Utilization**	**Leafhopper Shannon diversity mean (±SE)**	**Leafhopper abundance mean (±SE)**	**Grass specialist Shannon diversity mean (±SE)**	**N**
Control	1x	4.02 (±0.38)	72.58 (±7.33)	3.1 (±0.33)	12
forb rich	1x	3.19 (±0.15)	60.75 (±8.69)	2.42 (±0.17)	12
graminoid rich	1x	3.69 (±0.27)	87 (±10.67)	2.98 (±0.22)	12
Control	3x	3.06 (±0.26)	110.58 (±9.03)	2.58 (±0.19)	12
forb rich	3x	2.64 (±0.18)	80 (±9.81)	2.13 (±0.12)	12
graminoid rich	3x	2.58 (±0.14)	130.5 (±19.31)	2.38 (±0.1)	12
