# Supplementary material for: Disentangling direct and indirect effects of experimental grassland management and plant functional-group manipulation on plant and leafhopper diversity
Source: BMC Ecol. 2014 Jan 17;14:1. doi: 10.1186/1472-6785-14-1 (PMC3945068; doi:10.1186/1472-6785-14-1)
Supplement: Additional file 4: Figure S3 — Box plot showing the effects of herbicide application and cutting frequency on Shannon diversity of grass specialist leafhopper. [file 1472-6785-14-1-S4.pdf]

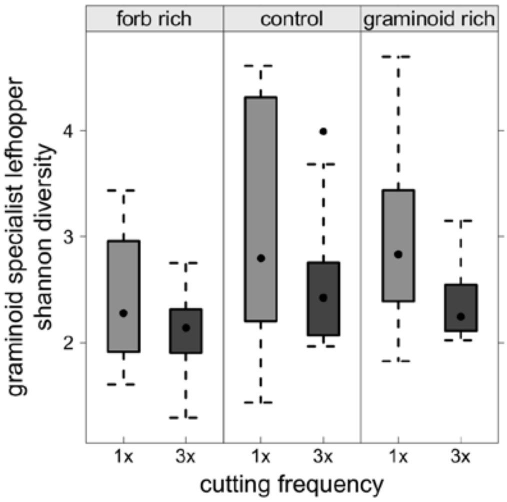

Figure S3: Box plot showing the effects of herbicide application and cutting frequency on Shannon diversity of grass specialist leafhopper. Most of the leafhopper species (28 out of 36) preferred grass as food source. The Shannon diversity of “grass specialist” leafhopper species was lower under three cuts/year (t= -3.34; P=0.001) and with herbicide altered plant composition (forb rich: t=-3.25; P=0.002, graminoid rich: t=2.33; P=0.023). We found no design effects on forb-preferring leafhoppers.
