# Supplementary material for: Disentangling direct and indirect effects of experimental grassland management and plant functional-group manipulation on plant and leafhopper diversity
Source: BMC Ecol. 2014 Jan 17;14:1. doi: 10.1186/1472-6785-14-1 (PMC3945068; doi:10.1186/1472-6785-14-1)
Supplement: Additional file 5: Figure S1 — Experimental design of the Grassman Experiment, showing the Latin rectangle of 12 treatments in 6 replications. Gra- = graminoid reduced plots (=forb enhanced); Forb- =forb reduced (=graminoid enhanced); Con = Control (no herbicide application). The grey area around and between the plots is mown monthly. Plot size 15 m x 15 m, space between plots 3 m, between blocks 5 m [file 1472-6785-14-1-S5.pdf]

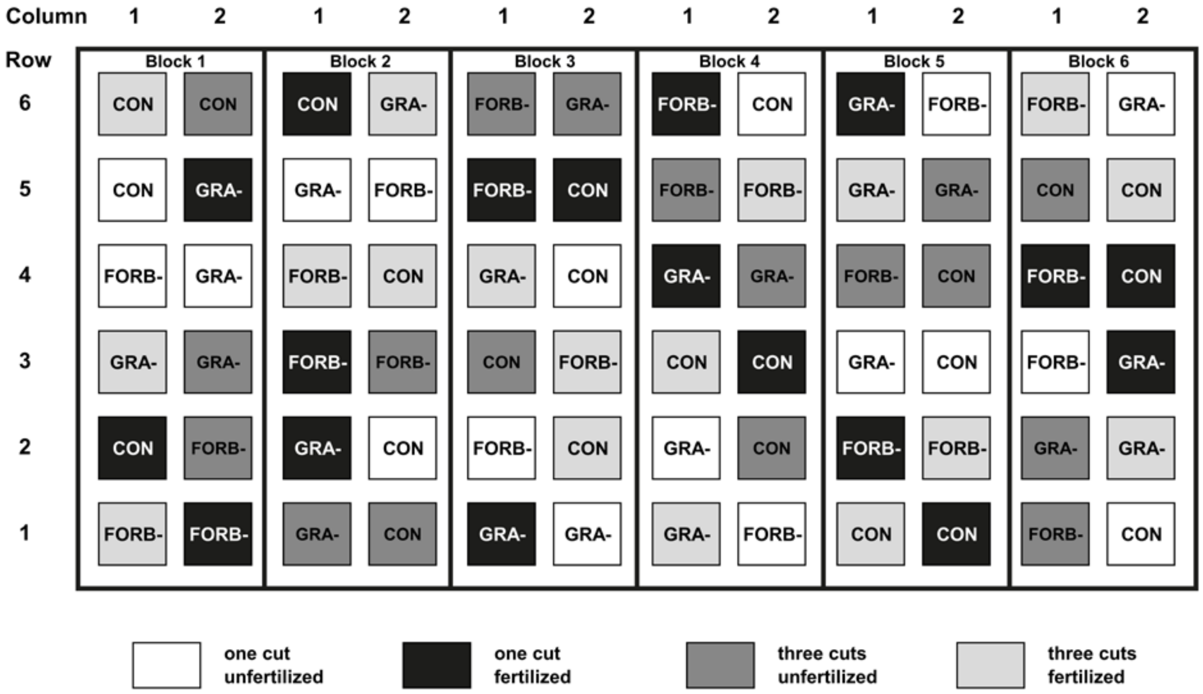

Figure S1: Experimental design of the Grassman Experiment, showing the Latin rectangle of 12 treatments in 6 replications. Gra- = graminoid reduced plots (=forb enhanced); Forb- =forb reduced (=graminoid enhanced); Con = Control (no herbicide application). The grey area around and between the plots is mown monthly. Plot size 15 m x 15 m, space between plots 3 m, between blocks 5 m.
