# Supplementary material for: Disentangling direct and indirect effects of experimental grassland management and plant functional-group manipulation on plant and leafhopper diversity
Source: BMC Ecol. 2014 Jan 17;14:1. doi: 10.1186/1472-6785-14-1 (PMC3945068; doi:10.1186/1472-6785-14-1)
Supplement: Additional file 6: Table S1 — F and p-values of glm’s testing for significant effects of sampling method in combination with design treatments. [file 1472-6785-14-1-S6.pdf]

Table S1: F and p-values of generalized linear models testing for effects of sampling method in combination with design treatments.

	Sampling		FG		Cutting		Sampling : FG	
	**F**	**(*p*)**	**F**	**(*p*)**	**F**	**(*p*)**	**F**	**(*p*)**
**Leafhopper species richness**	197.37	(<0.001)	7.68	(<0.001)	n.s.		n.s.	
**Leafhopper Shannon diversity (eH´)**	55.43	(<0.001)	6.95	(0.001)	n.s.		n.s.	
**Leafhopper abundance**	342.06	(<0.001)	2.08	(0.125)	10.21	(0.001)	6.38	(0.002)
