# Supplementary material for: Disentangling direct and indirect effects of experimental grassland management and plant functional-group manipulation on plant and leafhopper diversity
Source: BMC Ecol. 2014 Jan 17;14:1. doi: 10.1186/1472-6785-14-1 (PMC3945068; doi:10.1186/1472-6785-14-1)
Supplement: Additional file 7: Figure S2 — Comparison of the two different sampling methods in combination with functional group manipulation and cutting frequency. (a) leafhopper species richness; (b) leafhopper Shannon diversity (eH´); (c) leafhopper abundance. [file 1472-6785-14-1-S7.pdf]

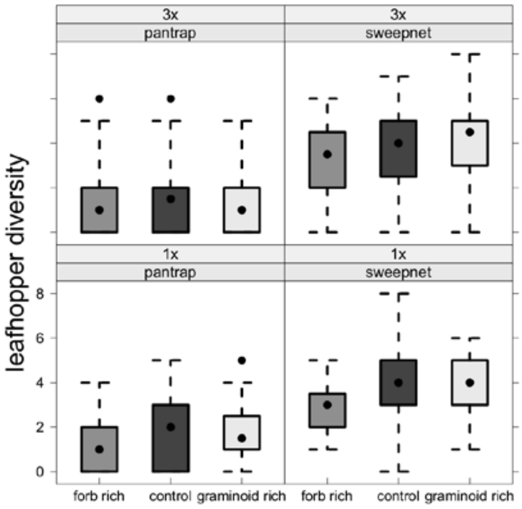

a)

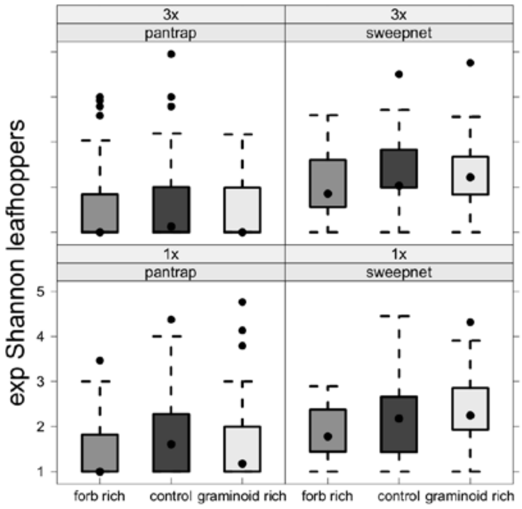

b)

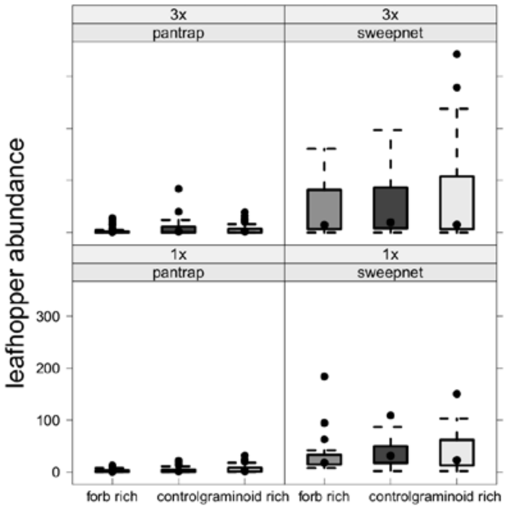

c)

Figure S2: Comparison of the two different sampling methods in combination with functional group manipulation and cutting frequency. (a) leafhopper species richness; (b) leafhopper Shannon diversity ($e^{H'}$); (c) leafhopper abundance.
