# Supplementary material for: Disentangling direct and indirect effects of experimental grassland management and plant functional-group manipulation on plant and leafhopper diversity
Source: BMC Ecol. 2014 Jan 17;14:1. doi: 10.1186/1472-6785-14-1 (PMC3945068; doi:10.1186/1472-6785-14-1)
Supplement: Additional file 8: Table S3 — ANOVA-table: Leafhopper abundance vs. Design. [file 1472-6785-14-1-S8.pdf]

Table S3: Sequential analysis of variance table from generalized linear model on leafhopper abundance vs. design variables

	Df	Deviance	Resid.DF	Resid.Dev	F	Pr(>F)
NULL	71	1542.32				
as.factor(row)	5	202.67	66	1339.65	4.8742	<0.001
as.factor(block)	5	419.38	61	920.28	10.0861	<0.001
FG manipulation	2	194.39	59	725.89	11.6877	<0.001
cutting frequency	1	236.7	58	489.19	28.4631	<0.001
